# Malnutrition and associated factors among children of adolescent mothers attending a tertiary hospital in Uganda

**DOI:** 10.4314/ahs.v24i4.51

**Published:** 2024-12

**Authors:** Geraldine Basanyukira, Violet Okaba, Elizabeth Kiboneka, Sarah Kiguli

**Affiliations:** Department of Paediatrics and Child Health, School of Medicine, Makerere University College of Heath Sciences, P.O.BOX 7042 Kampala, Uganda

**Keywords:** Malnutrition, teenage mothers, children of teenage mothers, Uganda, nutrition status

## Abstract

**Background:**

Children bearing children” emphasizes vulnerability of both parties involved. Children of adolescent mothers are prone to poor health outcomes, undernutrition inclusive.

**Objective:**

To assess the nutrition status and factors associated with under nutrition among children aged one to twenty-four months, of adolescent mothers(COAM) attending Mulago Hospital.

**Methodology:**

A cross-sectional study conducted at pediatric department of Mulago Hospital. COAM were recruited consecutively following consent. Mother-baby pair underwent history, physical exam, anthropometry and HIV testing. Data was extracted by a standardized questionnaire, entered into Epidata 3.1 and analyzed with SPSS Version19.

**Results:**

The prevalence of stunting, wasting and underweight was 32%, 31% and 27% respectively. Age above twelve months[OR 4.2CI 95 % (2.12 -8.32)p<0.001], partner lack of financial support[OR2.093CI 95%(1.1- 3.97)p<0.024], chronic illness[OR 3.901CI 95%(2.21-6.87)p<0.001], low birth weight[OR3.537CI 95%(1.5- 8.1)p<0.003], rural residence[OR 2.65CI95%(1.23-5.07)p 0.013] were associated with stunting. Factors associated with wasting: partner lack of financial support[OR 2.0CI 95%(1.06- 3.78)p 0.032], prematurity[OR 2.115CI 95%(1.02-4.35)p0.042], employed mother[OR 2.174CI 95%(1.18- 3.97),p 0.012] and inadequate antenatal care visits[OR1.83CI 95%(0.97-5.55)p 0.031].

**Conclusion:**

The high burden of under nutrition among COAM is of concern to parents, community and policy makers. Therefore, delaying teenage pregnancies, education on proper nutrition practices will reduce on the high burden.

## Introduction

Nutrition status as defined by the extent to which nutrients are available to meet the metabolic needs, encompasses normal nutrition status, under nutrition and over nutrition. Of concern is under nutrition, a serious public health problem in developing countries that has been linked to substantial increase premature deaths.

Of related concern, is the large proportion of adolescent mothers in sub-Saharan Africa and the link between adolescent motherhood and poor child health outcomes, under nutrition inclusive. In Uganda, the proportion of adolescent girls who have started child bearing has decreased by 18% over a period of 21 years (from 43% in 1995 to 25% in 2016). However, a pregnancy rate of 25% among adolescent girls in a fairly not so high population of Uganda, is still of great statistical impact^1^.

As per WHO, an adolescent is defined by age between 10-19 years of age. Adolescence being a transition stage, girls are growing faster than at any time after their first year of life with high nutrition demands. Therefore, having a child at this point escalates the child's risk of being undernourished due to the shared high nutrient demands by both mother and child^2^.

An Indian study among socially excluded groups, found that teenage mothers faced significant levels of stress that led to bad health outcome^3^. India being one of the countries with high rates of under nutrition in the world, a cross sectional study of a nationally representative sample noted a high prevalence of underweight and stunting in COAM at 47% and 41.8% respectively^4^. In Africa, work done in Tanzania, Sierra Leone equally noted a high prevalence of under nutrition of 50% and 90% (Gomez classification) respectively among COAM^5^-^7^. Under nutrition commonly affects children after six months of age. However, with the various factors surrounding adolescents including inadequate breastfeeding practices, under nutrition among their children is bound to set in earlier than six months of age and thus the inclusion of children 1 to 24 months. Under nutrition makes children more susceptible to infection leaving them with fewer reserves to recover from illnesses, thereby greatly impacting on their neuro cognitive potential and development^8^, social interaction and eventually their future capability in the various fields of life^9^. The lack of published data on the nutrition status and factors associated with under nutrition among children of adolescent mothers further justified the need to conduct this study in our setting which has high rates of teenage pregnancy.

## Materials and methods

### Study site/setting

The study was carried out in Mulago National Referral Hospital, located North West of Mulago hill in Kampala city with 1500 bed capacity. Being a National referral hospital, it receives patients from all over the country with the greater percentage from Kampala and neighboring district of Wakiso. The children were recruited from the Pediatric Outpatient Clinic, Acute Care Unit and Young Child Clinics as they are all entry points for adolescent mothers and their children. The clinics are proximal to the main screening area of the hospital. The pediatric outpatient and young child clinics operate Monday to Friday between 8am to 2pm, while the acute care unit operates daily for 24 hours with pediatricians, residents, nurses among others. The Pediatric Outpatient clinic mainly handles outpatients with uncomplicated illness but also screens and refers the critically ill to be managed in Acute Care Unit, the emergency pediatric unit. The bigger percentages of those seen in Acute Care Unit are managed as inpatient. The Young Child Clinic handles well children from birth to two years of age. Immunization and prevention of mother to child transmission of HIV are the main services offered. An average of six, five and four adolescent mothers-child pairs are seen daily at Young Child Clinic, Pediatric Outpatient clinic and Acute Care Unit respectively.

### Study participants and sampling procedures

We included children aged 1 to 24 months of adolescent mothers whose parents gave informed consent. children with severe limb disability whose anthropometry couldn't be taken by standard measures and mothers who did not know the child's date of birth were excluded. Consecutive enrollment was done until sample size of 348 was attained. Sample size was arrived at using the modified Keish Leslie formula for cross sectional studies as below.

### Sample size calculation

Determination of the sample size (n) was derived using the Kish Leslie formular (1965).


n=n1  1+n1  N

Where n = Zα/22pq

e2Zα/2 - The standard normal variate corresponding to the 95% confidence interval = 1.96, N − 600. The level of precision that's 5% or 0.05, P 34.7% (outcome condition) that was derived from similar study in South Africa^6^. From the computation the sample size was calculated at 348.

### Study procedure

The mother and child's ages were captured by the registration officers at the different study sites. This was done by self-report and then tallied with the reported date of birth.

The study was then introduced and explained to the mother. Following attainment of informed consent, the mother was then asked for consent for an HIV test. The risks to the child, rights of the patient as well as voluntary participation in the study were given to the mother to read through and explanation given where they did not understand. A detailed nutrition history of the child was taken and relevant physical examination performed to look for edema, signs of wasting and other obvious clinical manifestations of micronutrient deficiency like angular stomatitis, rachitic rosary, corneal ulcer or clouding. This was done by the principle investigator and the research assistants, (nutritionist) who had received further training in identifying these particular signs and symptoms in line with the study objective. The anthropometric measurements taken were weight, length and MUAC for children above six months. While nutrition status may not be observed directly, there are observable indicators i.e. biochemical, clinical and anthropometric. The anthropometric measurements indices were obtained using WHO growth references 2007 with reasons below: these are the most recently revised growth charts, the hospital where study was conducted uses similar charts, but also they were used in most of the similar studies referenced in this work. Weight was measured with at most accuracy using a two in one digital weighing scale (SECCA). Two readings were taken and their average recorded to the nearest 0.1kg. Length was measured with at most accuracy using a wooden SECCA stadiometer. Two readings were taken and their average recorded to the nearest 0.1cm. The different anthropometric parameters were then computed. (Weight for age, weight for length and length for age for child). Mother's weight and height was taken and BMI for age calculated using WHO Z scores. The Composite Index Anthropometric Failure(CIAF) as used in an Indian study, is said to be a better tool in assessment of malnutrition as it encompasses all the three individual nutrition indices^17^. However, it is said to exaggerate the statistics compared to the conventional nutrition indices. The mother's blood samples for HIV testing was then collected by needle prick and analyzed by determine rapid diagnostic test. The HIV exposed children; Iml of blood from an EDTA vacutainer was taken from a superficial vein and sent for DNA PCR. HIV tests were repeated even among those that had reported to be positive as none of the mothers had any documented proof of the result done previously. The outcome dependent variable was nutritional status as determined by Z scores for different parameters of weight for length, weight for age and height for age.

The independent variables were assessed using a pretested questionnaire. The tool was semi structured with both open and closed ended questions. The research tool captured data on socio-demographic characteristics, health care practices, food storage and community practices of the study participants. Quality control was ensured in this study. A pilot study was conducted for three days where research assistants were trained on how to take anthropometry, HIV testing and identifying clinical signs of micronutrient deficiency. Weighing scales and stadio meters were standardized with regular calibration. Weighing scales were calibrated to zero after every measurement. Research assistants were fluent in both English and Luganda, the local language in the region. Completed questionnaires were checked by principle investigator for accuracy, completeness, consistency before leaving study site.

### Data management and analysis

Data was then entered into Epidata/Epiinfo and then transferred to SPSS by a stastician. Data audits using frequency distributions and cross tabulation was performed to detect missing out of range and illogical values. data cleaning, range and consistence checks were run for each variable to identify values. In cases where it was not possible to correct errors in the data, a missing code was assigned. For storage and retrieval of data, questionnaires and all data entry forms was securely stored in order to maintain confidentiality at the end of data collection. Univariate analysis was conducted to determine prevalence of under nutrition in children of adolescent mothers. Findings were presented using frequencies and proportions. Bivariate analysis was conducted for factors associated with under nutrition among the children and the results summarized in bivariate tables. Tests for statistical significance were conducted using chi-square tests and p-values. Multi-variable analysis using binomial logistic regression was then conducted to adjust for possible confounding to develop a model for predictors of under nutrition in children born to adolescent mothers.

### Ethical considerations

Ethical clearance was obtained from the Makerere University School of Medicine Institutional Review Board. Informed consent was sought from mothers to participate in the study and also have their HIV status determined. Mothers less than 18 years equally gave consent as they are categorized as emancipated minors. The children found to be malnourished were referred to the nutrition unit of the hospital for standard care. HIV exposed infants were started on clotrimoxazole prophylaxis and their DNA PCR results followed up.

## Results

### Study profile

The study was conducted between September 2016 to March 2017. A total of 348 adolescent mother–child pairs were recruited. 129 (37%) of the study participants were enrolled from Acute Care Unit, 124 (35%) from Young Child clinic and 95 (27%) from Pediatric Outpatient clinic.

**Figure 1:**
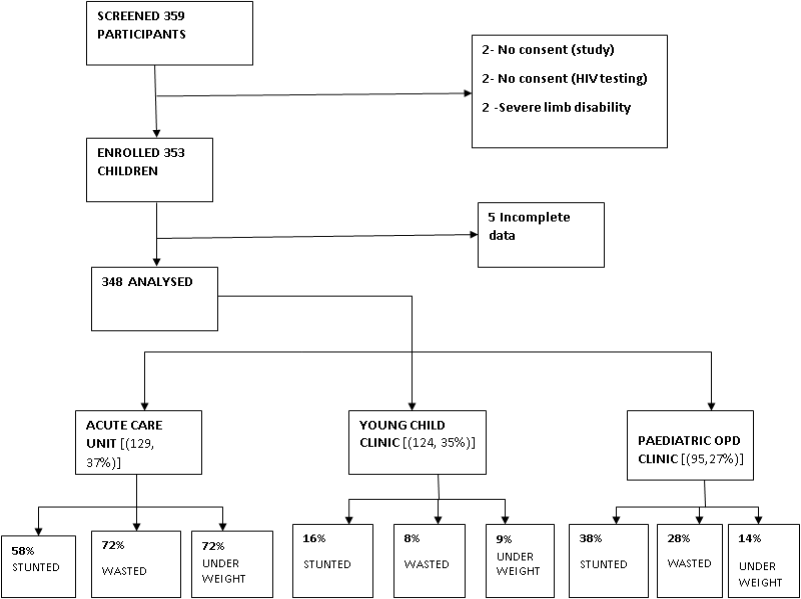
Study chart of the study participants

### Characteristics of study participants

Of the total children recruited, 177(50.9%) were male, while 171(49.1%) were female giving a male to female ratio of 1:1. The median age for the children in the study was 7.7 months. The rest of characteristics are summarized in table 1.

**Table 1:** Characteristics of children aged one to twenty-four months of adolescent mothers attending Mulago Hospital

Variable	Frequency (N=348)	Percentage %
**Age (months)**		
≤6	173	49.71
7-12	105	30.17
>12	70	20.11
**Sex of child**		
Male	177	50.86
Female	171	49.14
**Birth order**		
1	322	92.53
2-3	26	7.47
**District**		
Kampala	242	69.54
Wakiso	72	20.69
Others	34	9.77
**Residence type**		
Rural	39	11.21
Urban	309	88.79
**People in household**		
2-3	177	50.86
4-5	102	29.31
>5	69	19.83
**HIV Status (child)**		
HIV Exposed positive	6	1.72
Negative	308	88.51
HIV Exposed negative	34	9.77

### Characteristics of parents

The median age of the mother was 18.3 years and 24.7 years for the fathers. In contrast, only 10(3%) of the fathers were equally adolescents. Majority of mothers were unemployed at 79% and 59% had attained secondary education. The rest of characteristics are summarized in table 2.

**Table 2:** Socio-demographic characteristics of the adolescent mothers attending Mulago hospital

Variable	Frequency (N=348)	Percentage %
**Mother's age**		
<18 years	55	15.80
18 years	104	29.89
19 years	189	54.31
**Marital status**		
Single	115	33.05
Married/cohabiting	203	58.33
widowed/separated/divorced	30	8.62
**Employment status**		
Employed	34	9.77
Self-Employed	39	11.21
Un-Employed	275	79.02
**Mother's education level**		
None	9	2.59
Primary	114	32.76
Secondary	206	59.20
Tertiary/university	19	5.46
**Stay with biological father**		
Yes	193	55.46
No	155	44.54
**Partner's age**		
≤19	10	2.87
20-25	219	62.93
>25	119	34.20
**Alcohol consumption**		
Yes	44	12.64
No	304	87.36
**HIV status (mother)**		
Positive	40	11.49
Negative	308	88.51

### Maternal and child health characteristics of participants

Of the 331(95%) mothers that attended antenatal care, only 57% of them had attended the recommended number of at least 4 times. The rest of characteristics are summarized in table 3.

**Table 3:** Pregnancy outcomes and medical characteristics among children of adolescent mothers attending Mulago hospital

Variable	Frequency (N=348)	Percentage
**Place of delivery**		
Health Unit	322	92.53
Home	21	6.03
Others	5	1.44
**Birth Outcome**		
Term	296	85.06
Preterm	52	14.94
**Birth weight (kg)**		
<2.5	50	14.37
2.5-4.0	288	82.76
>4.0	10	2.87
**Attended antenatal**		
Yes	331	95.11
No	17	4.89
**Number of visits**		
<4 visits	141	42.6
≥4 visits	190	57.4
**Exclusively Breastfed**		
Yes	314	90.23
No	34	9.77
**Duration of exclusive** **breastfeeding(Mean SD=4mo)**		
<6months	197	62.74
6months	102	32.48
>6months	15	4.78
**up to date immunization**		
Yes	296	85.06
No	52	14.94
**Chronic medical illness**		
Yes	94	27.01
No	254	72.99

### Individual dietary diversity

This was calculated for children above 6 months of age, derived from feeds consumed a day prior to the interview. A total of 143 (73%) of children scored at most 50% food diversity while only 54(27%) respondents scored above 50%

### Micronutrient deficiency (clinical) among children of adolescent mothers

Of the 72 children who had clinical features of micronutrient deficiency, 48(66.7%) had clinical features of iron deficiency (angular chelitis, chelosis, stomatitis), 1(1.4%) had Vitamin A deficiency, 2 (2.8%) vitamin D deficiency (rachitic rosary, widened wrists).

### Nutrition status among children aged one to twenty-four months of adolescent mothers

Of the total 348 children recruited, 253 (72%) had a normal weight for age, 240 (69%) had normal weight for height parameters while 236 (68%) had normal height for age indices. According to WHO standards, the global acute malnutrition rate was 31% with 23% being severely wasted. The study found 112(32%) of the children to be stunted with 11.78% severely stunted. While 27.3% of the children were under weight and 17.82% severely underweight, less than 1% were found to be overweight. Importantly, the male children were more stunted and underweight compared to their female counterparts who were more wasted.

### Mother's nutrition status

The BMI for age among the mothers was computed using the WHO Z scores.

The study found that majority of the mothers had a normal BMI for age at 97%, none of the mothers was thin, nine (2.5%) were severely thin, while only one (0.29%) was obese.

### Multivariate analysis for wasting

All variables significant at bivariate p<0.2, were considered for multivariate analysis. Factors associated with wasting include: lack of financial support from father (OR=2.0, p= 0.32), preterm birth (OR= 2.115, p= 0.042). The rest of the variables are summarized in table 5 below.

**Table 5:** Multivariate analysis for the factors associated with wasting among the children aged one to twenty-four months of adolescent mothers attending Mulago hospital

VARIABLES	Unadjusted odds ratio(95% CI)	p- value	Adjusted odds ratio(95% CI)	p-value
**District**				
Kampala	1		1	
Wakiso	2.19(1.25-3.82)	0.006 *	1.873(1.00-3.49)	0.049 *
Others	5.95(2.77-12.76)	<0.001*	5.518(2.38-12.80)	<0.001*
**Employment (Mother)**				
Employed	2.14(1.27-3.59)	0.004*	2.174(1.18-3.97)	0.012 *
Un-Employed	1		1	
**Mother takes alcohol**				
Yes	1		1	
No	0.49(0.25-0.93)	0.029*	0.569(0.27-1.19)	0.134
**Family planning**				
Yes	1		1	
No	1.77(1.06-2.94)	0.028*	1.704(0.94-3.08)	0.079
**Father's financial support**				
Yes	1		1	
No	3.11(1.81-5.39)	<0.001*	2.000(1.06-3.78)	0.032 *
**Birth Outcome**				
Term	1		1	
Preterm	2.16(1.18-3.94)	0.012*	2.115(1.02-4.35)	0.042 *
**ANC visits**				
<4 visits	2.19(1.35-3.52)	0.001*	1.831(0.97-5.55)	0.031*
≥4 visits	1		1	
**Exclusively Breastfed**				
Yes	1		1	
No	2.14(1.04-4.34)	0.037*	2.315(0.96-5.54)	0.060

* ***Significant p values less than 0.05***

### Multivariate for stunting

All variables significant at bivariate p<0.2 for stunting were considered for multivariate analysis. Factors that were associated with stunting include: chronic medical illness (OR=3.901, p< 0.001), age above 12 months (OR=4.204, p < 0.001). The rest of characteristics are summarized in table 6 below.

**Table 6:** Multivariate analysis for the factors associated with stunting among the children aged one to twenty-four months of adolescent mothers attending Mulago hospital

Variables	Unadjusted odds ratio (95%CI)	P-value	Adjusted odds ratio(95% CI)	P-value
**Residence type**				
Rural	4.28(2.14-8.56)	<0.001*	2.650(1.23-5.07)	0.013*
Urban	1		1	
**Sex of child**				
Male	1		1	
Female	0.65(0.41-1.02)	0.066	0.712(0.42-1.21)	0.213
**Alcohol consumption**				
Yes	1		1	
No	0.52(0.27-0.98)	0.046*	0.676(0.32-1.75)	0.314
**HIV status (child)**				
HIV Exposed positive	10.4(1.19-90.2)	0.034*	1	
Negative	1		0.163(0.02-1.61)	0.121
HIV Exposed negative	0.53(0.23-1.28)	0.162	0.061(0.01-0.73)	0.028
**Father's Financial support**				
Yes	1		1	
No	2.12(1.23-3.67)	0.007*	2.093(1.10-3.97)	0.024*
**Birth Outcome**				
Term	1		1	
Preterm	2.44(1.34-4.44)	0.003*	1.801(0.79-4.10)	0.161
**Birth Weight (kg)**				
<2.5	3.31(1.78-6.12)	<0.001*	3.537(1.54-8.10)	0.003*
2.5-4.0	1		1	
>4.0	1.73(0.47-6.30)	0.404	0.553(0.12-2.66)	0.037*
**Chronic medical illness**				
Yes	3.52(2.14-5.78)	<0.001*	3.901(2.21-6.87)	<0.001*
No	1		1	
**Age of the child (months)**				
≤6	1		1	
7-12	1.47(0.86-2.53)	0.159	1.521(0.81-2.87)	0.196
>12	3.82(2.12-6.87)	<0.001*	4.204(2.12-8.32)	<0.001*

* ***Significant p values less than 0.05, chronic illness (cardiac disease and sickle cell anaemia)***

## Discussion

### Prevalence of under nutrition

The prevalence of stunting in COAM was 32%. This is high but comparable to the prevalence of 29% in the National UDHS statistics of Uganda (2016) carried out in the general paediatric population among children under five^18^. Finding a prevalence in COAM almost equating to the general paediatric population, justifies the burden and increased risk of under nutrition among COAM by virtue of their associated risk. The prevalence of stunting of 32% was similar to the prevalence reported in the South Africa study at 34.7% among a similar study population, of COAM^6^.

However, the prevalence of stunting at 32% in this study was comparably lower than that found in the national survey of India at 41.7%^4^ among COAM. India having the highest burden of under nutrition in the world and the large study population used of up to five years in the Indian study could explain the high statistics. Further still, under nutrition is more common in children above two years due to inconsistence with foods availability unlike those less than two years that additionally have breast milk as part of required diet which is cheap and more accessible.

Unlike stunting, a chronic marker of under nutrition, wasting is an acute maker of under nutrition of which the study obtained a prevalence of 31%. This is very high compared to the national statistics of 4 % in the general paediatric population^18^. A hospital study site with majority of participants recruited from Acute Care Unit, where the nutrition state of child may be affected by an acute illness can explain the rather high statistics. However, the high prevalence further depicts the increased risk of under nutrition in COAM as compared to the general paediatric population. Compared to the Indian study, which reported a prevalence of 17%, the prevalence of wasting in this study was high at 31%^4^. In the Indian study, work had been carried out among a wellness clinic set up which is likely to have fewer children with wasting an acute marker of under nutrition as compared to this study where participants had been recruited from hospital set up.

In this study, the male child was more likely to be stunted and underweight as compared to the female child. This is consistent with majority of work that has been done in under nutrition^4^, which has explained it by: explorative nature of boys, vigorous exercise and being away from home unlike their female counterparts that may take part in food preparation process. However, the age category considered in this study may less likely fit with the above explanations since these children are almost entirely dependent on their mothers which then questions the likelihood of a possible genetic influence among boys.

### Factors associated with under nutrition among children of adolescent mothers

In this study, mixed feeding was associated with being wasted, which is in line with findings by Bailey in Brazil among a similar study population^19^, where short duration of exclusive breastfeeding (three months) was associated with being wasted

Unlike in India where maternal malnutrition was greatly associated with under nutrition^4^, this study found only 9 (2.5%) mothers having severe thinness with the majority at 97% having normal BMI for age.

Having an HIV positive child increased their risk of being wasted which was however not significant at multivariate. Of the forty HIV exposed infants, only six were diagnosed positive. This could be attributed to the role out of Elimination of Mother to Child Transmission measures in Uganda since 2013 and thus the low transmission rates seen in this study.

Residing in a urban setting was protective against wasting and stunting at multivariate analysis, which is similar to work in India^4^. This emphasizes the likelihood of poor social economic status, food insecurity but also higher likelihood of illness among children that reside in rural areas that may directly or indirectly lead to under nutrition.

Children not receiving financial support from the father was associated with increased chances of being stunted and wasting at multivariate analysis. This was in keeping with work done in Brazil^20^ among a similar study population which justifies the role of paternal support in the wellbeing and nourishment of both mother and child.

Children above 12 months of age were more likely to be stunted compared to their counterparts below 12 months. Similarly, in Kenya^21^ children in their second year of life, were more likely to be stunted. Children below one year are mainly dependent on breastfeeding which is cheap and affordable unlike their counterparts above 12 months who mainly feed on household diets, these are usually difficult to consistently receive in the right quantities coupled with the limited knowledge of the desired foods needed for adequate growth.

Having a chronic medical illness was associated with being stunted which is in keeping with work done in general paediatric population. This study found cardiac disease being the most reported chronic illness followed by sickle cell anaemia at 2% and 1% respectively. Children with chronic illness have reduced food intake, increased metabolic demands which may eventually lead to stunting.

Contrary to what has been found in some studies, having an un employed mother was protective against being stunted. This could be attributed to majority of employed mothers reported leaving their children with the neighbours and therefore didn't supervise their children's feeding and only got involved in caring and feeding of their children for a few hours of the day.

Unlike their babies, biggest percentage of mothers had a normal BMI for age. Therefore, having normal nutrition status for most of the adolescent mothers may exclude genetic predisposition to undernutrition of their babies but rather be attributed to by environmental factors/constraints in care of the baby. This is different from work done in India where maternal malnutrition contributed to their child's malnutrition^15^.

Finally, having a low birth weight was associated with stunting while attending antenatal care services less than the recommended four times was associated with being wasted. Children with low birth weight may have difficulty achieving their catch up weight in the presence of limited financial resources. This is turn explains the role of adequate prenatal care in determining the nutrition status of children.

## Limitations

Due to financial constraints, this study did not investigate for laboratory evidence of micronutrient deficiencies which could impact poorly on growth,

Lack of documented proof for age of the mothers like birth certificates, as we relied more on mother's self-reported age tallied with the year of birth.

A hospital study could have overestimated the burden of wasting

## Conclusion

The study found a high burden of Under nutrition in children of adolescent mothers, prevalence of stunting, wasting and underweight at 32%, 31% and 27% respectively. These high figures further depict the vulnerability of this particular group of children due to the enormous challenges faced by their mothers.

The factors found to be associated with stunting were rural residence, lack of financial support from father, preterm births, having a chronic illness, child's age above 12 months. Having an employed mother, attending antenatal care less than the recommended four times, lack of financial support from the mother and preterm birth was associated with wasting of the child.

Poor nutrition state negatively impacts the child's growth, school attendance and performance^22^ development and wellbeing with potential to poor health state even as adults.

Therefore, advocating for contraceptive accessibility to teenagers coupled with sensitization to ending teenage pregnancies will not only reduce the escalating teenage motherhood but also reduce on occurrence of adverse health outcomes to their babies and associated mortality.

## Recommendation

1. Basing on the statistics above, there is need for routine screening for under nutrition in children of adolescent mothers and continued provision of health education towards good health practices especially among those at increased risk.
2. Adolescent mothers should be encouraged to attend the required antenatal care visits and provision of adolescent friendly services at antenatal care points which may improve birth outcome and further reduce the prevalence of under nutrition.
3. A community study preferably a comparative study among adult and adolescent mothers will be more informative of the true picture on the nutrition status but will also be able to identify the risk factors to under nutrition among either age category.

## Figures and Tables

**Table 4:** Nutrition status in children of adolescent mothers aged one to twenty-four months attending Mulago Hospital

		Number N	Prevalence %	Overall %
**Stunting** (HFA)	Severe (<-3SD)	41	**11.78**	**32.2**
	
	Moderate (-3SD<-2SD)	71	**20.40**	
	
	Normal (>-2SD)	236	67.82	
**Wasting**				**31**
(WFL)	Severe(<-3SD)	82	**23.56**	
	Moderate (-3SD<-2SD)	26	**7.47**	
	Normal (-2SD<2SD)	240	68.97	
**Underweight**				**27.3**
	
(WFA)	Severe (<-3SD)	62	**17.82**	
	
	Moderate (-3<-2SD)	33	**9.48**	
	
	Normal (-2SD <2SD)	251 72.13		
	
	Over Weight (>2SD)	2	0.57	

* WHO Z score HFA height for age, WFA weight for length, WFL (weight for age*SD Standard deviation

## List of Abbreviations

BMI: Body Mass Index
COAM: Children of Adolescent Mothers
HIV: Human Immune-deficiency Virus
MAM: Moderated Acute Malnutrition
MDG: Millennium Development Goals
MUAC: Mid Upper Arm Circumference
SD: Standard Deviation
UBOS: Uganda Bureau of Statistics
UDHS: Uganda Demographic Health Survey
UNICEF: United Nations Children Emergency Fund
WF: Weight For Length
LFA: Height for Age
WHO: World Health Organization

## Author's information

GB: MBChB MMED(Mak). was a resident (student of masters in medicine Pediatrics and child health)at Makerere University College of Health Sciences by the time of the study, but now a pediatrician at Kawempe National Referral Hospital foe the past 4 years.

VO: MBChB.MMED(Mak), lecturer of pediatrics Makerere University, College of Health Sciences.

EK: Consultant pediatrician, Mulago National Referral Hospital.

SK: MMED, MAK, MHPE(MAASTRICHT), Professor of Pediatrics, Makerere University College of Health Sciences.

Contact information for the corresponding author

Name: Geraldine Basanyukira,

+256782856324 Email: gerib2004@yahoo.com
